# Malnutrition Affects the Urban-Poor Disproportionately: A Study of Nigerian Urban Children of Different Socio-Economic Statuses

**DOI:** 10.3390/children3040017

**Published:** 2016-09-23

**Authors:** Chukwunonso E.C.C. Ejike

**Affiliations:** 1 Department of Medical Biochemistry, Faculty of Basic Medical Sciences, Federal University Ndufu-Alike Ikwo, PMB 1010 Abakaliki, Ebonyi State, Nigeria; nonsoejikeecc@yahoo.com; Tel.: +234-08036066777; 2 Department of Biochemistry, Michael Okpara University of Agriculture, Umudike, PMB 7267 Umuahia, Abia State, Nigeria (affiliation at the time the research was conducted)

**Keywords:** children, malnutrition, socioeconomic status, thinness, overweight/obesity

## Abstract

Income inequality within the same place of residence may impact the nutritional status of children. This study therefore investigated the impact of income inequality on the nutritional status of children living in the same place of residence, using anthropometric tools. Children in four schools (Schools 1–4) within the vicinity of a housing estate in Umuahia, Nigeria, that charge fees making them ‘very affordable’, ‘affordable’, ‘expensive’ and ‘very expensive’, respectively, were recruited for the study. Thinness, overweight and obesity were defined using the Cole et al. reference standards. Thinness was present in 10.4% (13.0% of boys, 7.6% of girls); 20.4% (15.6% of boys, 27.3% of girls; and 0.7% (1.4% of boys, 0.0% of girls) of children in Schools 1–3, respectively; but absent in school 4. Only 3.7% (1.4% of boys, 6.1% of girls) and 5.6% (6.3% of boys, 4.5% of girls) of children in Schools 1 and 2, respectively, were overweight/obese. Conversely, 25.8% (18.9% of boys, 32.5% of girls) and 41.6% (38.8% of boys, 45.3% of girls) of children in Schools 3 and 4, respectively, were overweight/obese. The urban-poor (School 2) are clearly affected by malnutrition disproportionately.

## 1. Introduction

Malnutrition in children, which clinically manifest as underweight and overweight/obesity, is a major public health challenge in low and middle income countries (LMICs). In Nigeria, there are reports of high prevalence of both under- and over-nutrition, even in the same locality [1,2]. This double burden of malnutrition in societies experiencing nutrition transition calls for deliberate holistic health policy action to cater for the needs of populations at risk of nutritional challenges. This is important because malnutrition in children not only represents both a cause and a manifestation of poverty [3], but is also known to lead to somatic and psychological challenges, including irreversible intellectual deficits, throughout the life course [4]. Furthermore, malnourished children are more likely to enrol late in school, drop-out more and perform worse than their well-nourished counterparts [5]. 

Place of residence and mother’s educational attainment are established in the literature as the major socioeconomic status (SES) markers that contribute significantly to the direction of the nutritional status of a child [6]. Here, the urban/rural divide is reported to be responsible for the bulk of the variation in the nutritional status of children. The argument being that urban dwellers earn more money than their rural counterparts, and have better access to proper housing, water, drainage, and healthcare facilities [7]. Furthermore, given the nature of the jobs in the cities, educated mothers, who more readily understand nutrition information, are more likely to be clustered within the city, enhancing the health advantage their children have over those in the rural areas. Other authors have, however, argued that SES rather than place of residence is the more important factor that affects the nutritional status of children [8]. Here, attention is drawn to the case of urban slums and the consistent poor nutritional status of the children in such places reported by authors from different countries [6,7,9]. It has therefore been advocated that efforts at disaggregating data from places of residence along SES lines are required to appreciate better the dynamics of nutritional achievements in children [10]. Such understanding is required to drive appropriate health policy responses/actions in order to contain the epidemic of nutritional imbalances currently plaguing LMICs.

**Figure 1:**
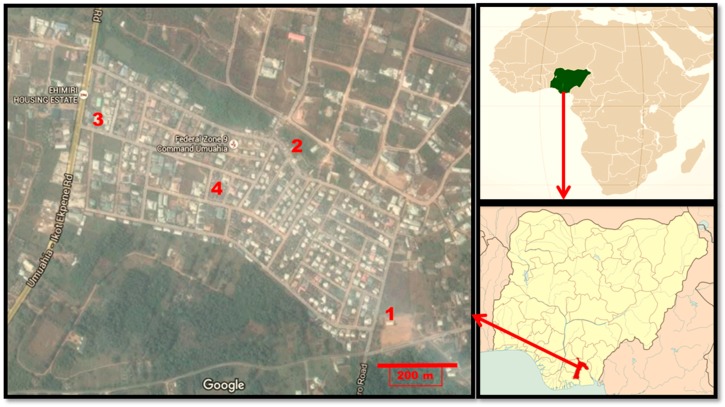
Map of Ehimiri housing estate, Umuahia, indicating the location of the studied Schools 1–4 and location on the maps of Nigeria and Africa. Adapted from Google Map. Scale bar = 200 m.

Previous studies in Nigeria have concentrated on rural/urban comparisons, thereby missing the income inequality and the attendant variation in the living standards of people living in either place of residence. This study investigates the nutritional status of children living in Ehimiri housing estate, a government-developed housing estate, located on the outskirts of Umuahia, Abia State, Nigeria. Due to wide variations in incomes, Ehimiri is home to families belonging to different SES groups. Interestingly, there are four different primary schools within 400 m from the centre of the housing estate (Figure 1). The four schools, designated Schools 1‒4, charge different fees described here (for the purposes of convenience) as very affordable (School 1), affordable (School 2), expensive (School 3) and very expensive (School 4). Whereas School 1 is a public school that caters for children from a village adjourning Ehimiri, Schools 2‒4 are private schools that cater for children living in the housing estate, depending on the financial position of their families. Considering that in Nigeria there is a pervasive ‘status mentality’ about the school children attend, the four schools reflect the SES of the families living in Ehimiri, which is essentially urban, with a rural village adjoining it. The four schools therefore present an opportunity to study the nutritional status of children living in the same place with different SES and of children living in a nearby village.

## 2. Materials and Methods

### 2.1. Subjects

Children aged 5‒12 attending any of the four nursery/primary schools within the vicinity of Ehimiri housing estate were potentially eligible for inclusion in this study. Written permissions to conduct the studies in the four schools were obtained. Thereafter, the parents/legal guardians of the children were written asking for permission to recruit their children/ward. Only children whose parents/legal guardians consented to their participation were eventually recruited. Other inclusion criteria for the study were (a) absence of any signs of overt illness and (b) non-use of any prescription (or other) drugs. A total of 460 children (52.6% males) were included. No honoraria were paid to participants.

### 2.2. Methods

The Schools 1–4 were used as a proxy for SES. Hence, children attending Schools 2–4 were taken to be the urban-poor, urban-middle income, and urban-rich children, respectively. Children in School 1 were simply taken as rural children. The ages (in months) of the children were obtained from their school records. Height was measured (with the child standing on bare feet) using a non-elastic measuring tape and adjusted to the nearest 0.5 cm. Weight was measured (with the child, bare footed, and with light clothing) using an electronic weighing balance (BF 214, OMRON Healthcare Europe BV, Hoofddorp, The Netherlands), to the nearest 0.1 kg. The same trained personnel took all measurements in all locations. From the height and weight data, body mass index (BMI) was calculated using the formula BMI = Weight (kg) / (Height (m))^2^. The weighing balances used were calibrated before use each morning according the manufacturer’s instructions. Each child was asked to provide information on (1) number of siblings; (2) means of transportation to school daily; and (3) whether or not they ate food snacks in school. The study protocol was prepared in accordance with the Helsinki Declaration and was approved by the Board of the Department of Biochemistry, Michael Okpara University of Agriculture, Umudike.

### 2.3. Definitions 

Thinness, overweight and obesity (irrespective of stage) were defined using the age and gender specific BMI cut-off points of Cole et al. [11,12].

### 2.4. Statistical Analysis

The variables were, for convenience purposes, stratified into two age ranges viz. 5–8 years old and 9–12 years old. Descriptive statistics were performed on the data generated (cumulatively and then based on the age ranges) and their results reported as mean ± standard deviation (SD) for continuous data, and as percentages for categorical data. Differences between means for continuous data were compared using one-way ANOVA analysis followed by least significant difference (LSD) post-hoc multiple comparisons. Where necessary, differences between categorical data were compared using the Fisher’s exact test (2-sided). For all analyses, the significant threshold was fixed at *p* < 0.05. Data analyses were performed using the statistical software IBM-SPSS version 20.0 (IBM Corp., Atlanta, GA, USA).

## 3. Results

The height of the children, irrespective of sex and school, were statistically similar (*p* > 0.05). Among the females, those in School 3 were the heaviest (33.8 ± 8.8 kg) and they were significantly heavier than their counterparts in Schools 1 and 2 (29.1 ± 5.4 kg; *p* = 0.001 and 28.7 ± 7.6 kg; *p* = 0.010, respectively), but not those in School 4 (31.8 ± 10.3 kg). Akin to the pattern observed for the weight of the children, the mean BMI was significantly higher in Schools 3 and 4, compared to Schools 1 and 2 (*p* < 0.001 in each case). A similar pattern for body weight and BMI was observed for the males. The reported number of siblings decreased linearly from School 1 to School 4. Children in School 1 reported having significantly (*p* < 0.01) more siblings (5 ± 2 siblings for both males and females) compared the other schools; while their counterparts in School 4 reported having the least number of siblings (2 ± 1 siblings for both males and females) (Table 1).

**Figure 2:**
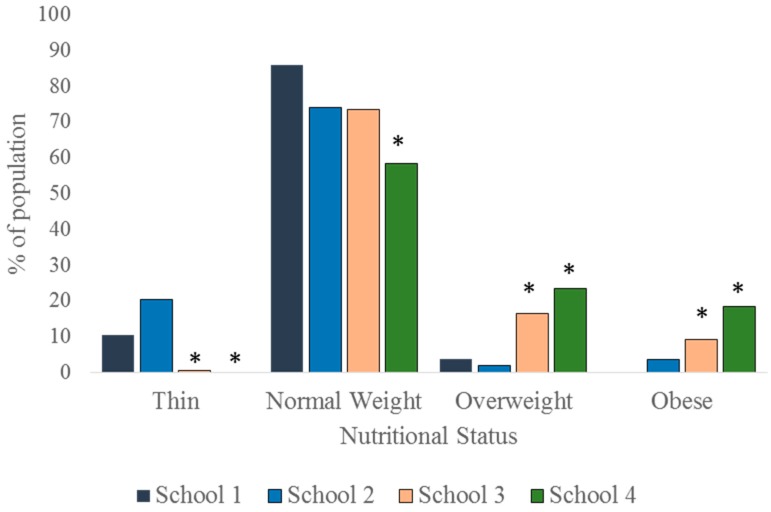
Nutritional status of the children, irrespective of age range and sex. *: *p* < 0.05.

When viewed without disaggregation along sex and age-range lines, it is seen that whereas none of the children in School 4 were thin, none of their counterparts in School 1 were obese. As much as 20.4% of children in School 2 were thin whereas the figure stood at 10.4% in School 1 and 0.7% in School 3. The prevalence of thinness (based on the World Health Organization (WHO) public health significance criteria) is therefore high in School 2, moderate in School 1, but low in Schools 3 and 4. The prevalence of thinness in School 1 was statistically similar to that of School 2 (*p* = 0.073). Thinness was, however, significantly less prevalent in Schools 3 and 4 (*p* = 0.010 and *p* = 0.002, respectively) compared to School 1. While as much as 41.6% and 25.8% of children in Schools 4 and 3, respectively, were either overweight or obese, only 3.7% and 5.6% of their counterparts in Schools 1 and 2, respectively, were in that category (Figure 2). Again overweight/obesity rate were similar in Schools 1 and 2 (*p* = 0.748), but significantly higher in Schools 3 and 4 (*p* < 0.001 and *p* < 0.001, respectively).

**Figure 3:**
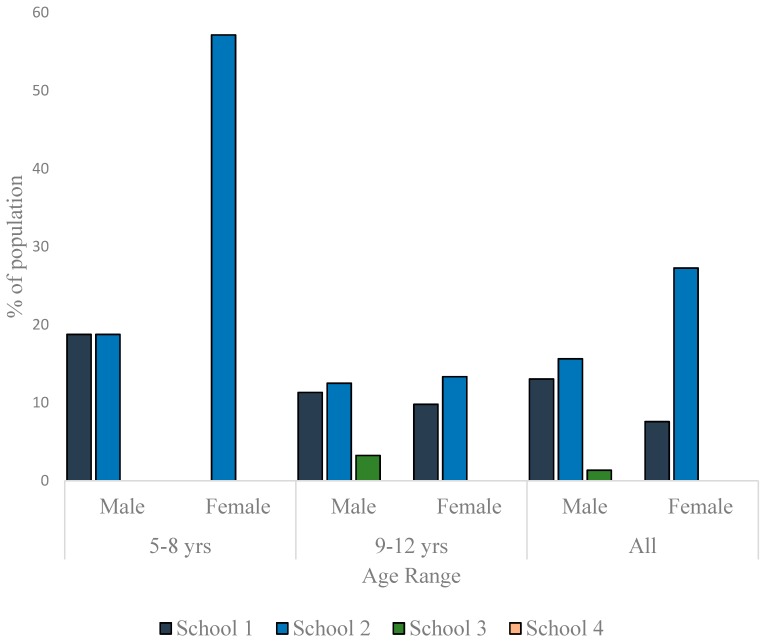
Prevalence of thinness among children in the studied schools.

**Figure 4:**
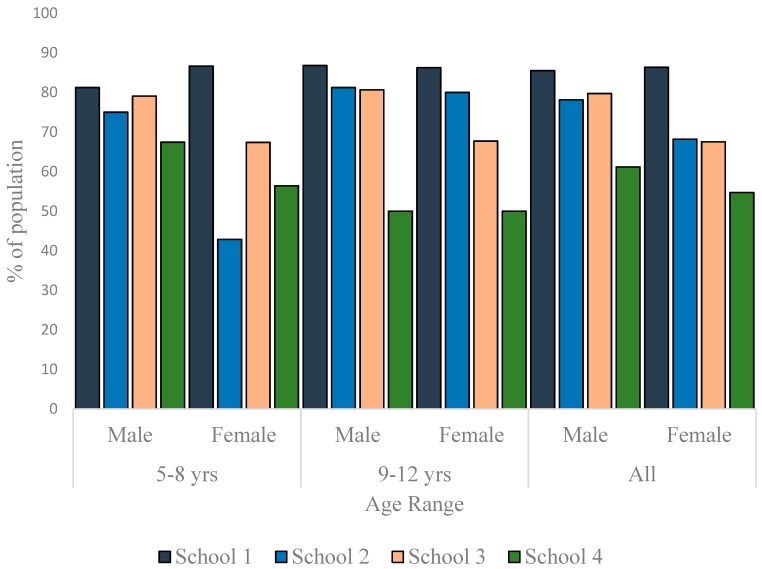
Distribution of normal weight children in the studied schools.

To appreciate the dynamics of the nutritional status of the children better, the data were disaggregated along sex and age-range lines and presented as per nutritional status. As much as 13% of the males and 7.6% of females in School 1 were thin. In School 2, the prevalence of thinness was 15.6% in males and 27.3% in females. In School 3, 1.4% of the males and none of the females were thin (Figure 3). The differences in the sex-specific prevalence of thinness were not significant in the schools where thin children were found (School 1: *p* = 0.358; School 2: *p* = 0.084; School 3: *p* = 1.000). There were more normal weight children in School 1 (85.5% boys, 86.4% girls) than in the other schools (Figure 4). Furthermore, whereas the proportion of normal weight children (irrespective of sex) was comparable in Schools 2 and 3, School 4 exhibited the lowest percentage of children with normal weight (61.2% boys, 54.7% girls) The proportion of normal weight children was significantly lower in School 4 population when compared to School 1 population (*p* = 0.008 and *<*0.001 for boys and girls, respectively).

**Figure 5:**
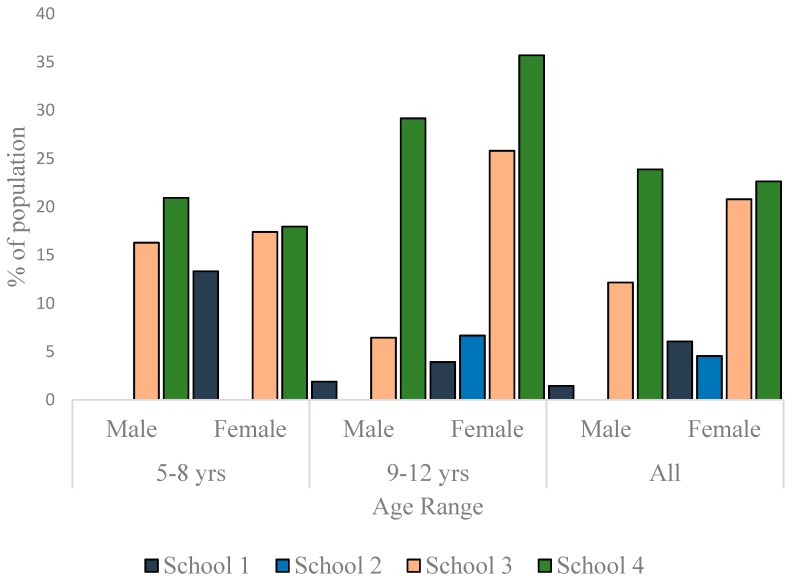
Overweight prevalence among children in the studied schools.

**Figure 6:**
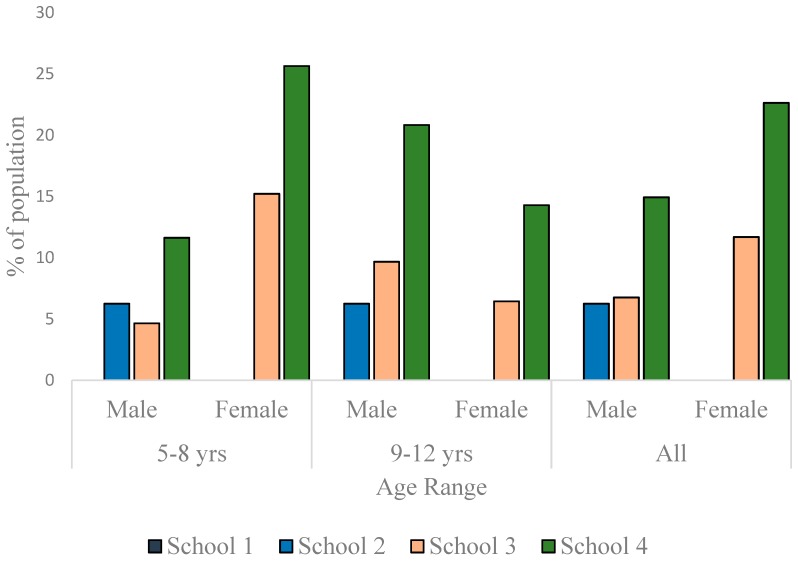
Prevalence of obesity among children in the studied schools.

As much as 23.9% (boys) and 22.6% (girls) in School 4 were overweight. In School 3, the prevalence of overweight was 12.2% for boys and 20.8% for girls. While there was no overweight boy in School 2, 4.6% of the girls were overweight. In School 1, the prevalence of overweight was 1.5% for boys and 6.1% for girls (Figure 5). Other than in School 4, the data showed a trend to have more girls overweight than boys, but the differences were not statistically significant in any school (*p* = 0.170 for School 1, *p* = 0.059 for School 2, *p* = 0.057 for School 3 and *p* = 1.000 for School 4). No child in School 1 was obese. In School 2, though no girl was found to be obese, 6.3% of the boys were obese. The prevalence of obesity in School 3 was 6.8% for boys and 11.7% for girls. In School 4, the prevalence of obesity was as high as 14.9% for boys and as 22.6% for girls. Though in Schools 3 and 4, girls were more obese than boys (Figure 6), the differences were not statistically significant (*p* = 0.335 and 0.207, respectively). The differences in the prevalence of obesity between the sexes was, however, significant in School 2 (*p* = 0.029).

Finally, we collected data for mode of transportation to school and snack food consumption by the children. All the children in School 1 reported that they went to and from school on foot daily. While 48.1% of the children in School 2 walked to and from school daily, the other 51.9% were driven to school in a vehicle. For School 2, there was no significant difference in the proportion of children based on means of arriving at school daily (*p* = 0.671). Only 4.0% and 1.3% of the children in School 3 and School 4, respectively, reported that they went to and from school on foot daily. In both schools, the rest of the children (proportionally significantly more; *p* < 0.001 for both cases) were driven to school in a vehicle. Only 8% of children in School 1 but all the children in Schools 2, 3 and 4 reported taking snack foods to school daily. Compared to School 1significantly more children went to school with snack foods in Schools 2, 3 and 4 (*p* < 0.001 in each case).

## 4. Discussion

The nutritional status of children is an indicator of the overall health condition of the population [13]. An earlier cross-sectional study in Umuahia, Nigeria, using the same diagnostic tool as used in this study reported a thinness prevalence of 17.3% in males and of 15.5% in females [2]. These values are closer to the values reported in Schools 1 (very affordable; rural) and 2 (affordable; urban-poor), but considerably higher than values reported for Schools 3 (expensive; urban-middle income) and 4 (very expensive; urban-rich). The said study also reported an overweight prevalence of 3.9% in boys and of 5.8% in girls, and an obesity prevalence of 0.3% in boys and of 1.0% in girls. Again, these figures are close to the values reported in Schools 1 and 2 but are clearly lower than values reported in Schools 3 and 4. This may be an indication that in Umuahia (and maybe in Nigeria as a whole), families that can afford to enroll their children/wards in expensive and very expensive schools (in other words, wealthy families) are in the minority, a reflection of the true economic situation in Nigeria. There are many other reports on the prevalence of obesity in children, but methodological differences and variations in sample characteristics make proper comparisons difficult. Nonetheless a recent 30-year review of studies on the prevalence of overweight and obesity in Nigerian children and adolescents showed that the prevalence of overweight was 5%–12% while that for obesity was 0.0%–5.8% [14]. The proportion of children overweight and obese in Schools 3 and 4 are therefore higher than previously reported values from cross-sectional studies conducted in Nigeria.

Though there are reports that increases in economic growth ultimately translate to improved nutritional status and health of the population [15], there is no consensus on the topic. It has been suggested that the impact of economic growth on the reduction in the prevalence of undernutrition in children, especially in developing countries, is negligible or non-existent [8]. This is because individual/household economies often vary from national per capita reports. The World Bank reports that undernutrition disproportionately burdens the poorest households who face the challenges of food and nutritional insecurity and lack access to proper healthcare [16]. The heterogeneity in the SES of families in urban areas of most LMICs is related to the observation that overweight/obesity prevalence is higher among wealthy urban residents, while the rural and the urban poor populations still battle with undernutrition [10]. Without prejudice to the above, income inequality is more pronounced in urban areas relative to rural areas [17] such that it is common to find both obese and thin children in the same urban area whereas obese children are rarely found in rural areas. The data presented here support the said differences in nutritional status of children based on the economic situation of their families. This study shows that thinness was almost absent in urban-middle income and urban-rich children (that is, children attending the expensive and very expensive schools, respectively). The data also reflect the disparities that exist in the nutritional status of ‘rural’ children versus their ‘urban-poor’ counterparts as they show proportionally more (though statistically similar) cases of thinness among children in poor families in the studied housing estate than among children from the nearby village (School 1). Furthermore, overweight/obesity increased with increasing SES. Implicit in the above is that the urban-poor children had a higher prevalence of thinness than their richer counterparts and concomitantly a higher prevalence of overweight/obesity than their rural counterparts. Similar findings have been reported in other African countries such as Kenya and Zambia [18,19]. In fact, variability in the nutritional status of children in sub-Saharan Africa has been attributed principally to intra-urban and intra-rural inequality in family income [17].

Interestingly, none of the children from School 1, a rural school, were obese whereas 41.6% of their counterparts in School 4 were overweight/obese. This may not be only due to reduced access to food for children in School 1, (as in fact thinness was moderate) but may be related to physical activity. Indeed, all the children in School 1 reported walking to and from school daily versus almost all the children in Schools 3 and 4 who were driven to and from school in vehicles. Many studies have shown that rural children engage in more physical activity compared to their urban counterparts [20,21]. The observations made here are therefore not isolated observations.

The finding that children with fewer siblings were more overweight/obese is in tandem with reports that parity substantially affects child health indices negatively [19]. This may be due to the fact that with a given income, fewer people would translate to larger shares of the income while larger family sizes will increase the chances of shortfalls, especially with respect to food. Implicit in this is the view that though higher SES is often related to high mother’s education, and educated mothers are more likely to adopt family planning strategies, and so have fewer children, people of the same income but different parity may still have differences in their overweight/obesity rates.

The consumption of processed snack foods by children is reportedly becoming common in LMICs [22]. This study corroborates this view and that of Banerjee and Duflo [23] who disagree with the view that poor families in LMICs lack sufficient income to purchase snack foods for young children. The data presented here show that the urban-poor buy snack foods for their children, even though it may be at the expense of proper nutrition at home. Interestingly, consumption of nutrient-poor diets and unhealthy snack foods contribute to both thinness and overweight [24]. It is therefore plausible that the poor nutritional status of the urban-poor reported here may be driven by the unhealthy snack foods and possible poor diets of the children. The lower number of siblings and being driven to and from school as found in the expensive and very expensive schools when viewed along the higher overweight/obese prevalence rates (and absence of thinness) clearly supports the impact of SES on the nutritional status of children. A lower SES often denotes large family size, poor education, poor living conditions, inadequate access to nutritious foods and safe water [7]. Conversely, access to cheap, nutritionally-poor foods and motorized transportation are classical underpinnings of a high SES, which drives overweight/obesity [25]. These observations support the patterns observed in this study. Clearly the urban-poor children have worse nutritional status than the children of the wealthier urban families but also than the children from a nearby village. Malnutrition indeed affects the urban-poor disproportionately.

This study is limited by a number of factors. First, only school-going children were included in the study; therefore, the study may not represent the general population. This is, however, attenuated by the fact that the design of the study was tied to its aim and as such it was inevitable to recruit only school-going children. Furthermore, school enrollment in the South-East of Nigeria is almost 100%. Second, the diagnostic criteria used for the study estimate the BMI of the children as a reflection of the equivalents at age 18. Generally, BMI may misclassify individuals as it does not account for the variations between fat mass, muscle mass, bone densities among others. Despite these factors, BMI is still a valid, widely used index that is very useful in epidemiologic studies. Third, classification of place of residence is often devoid of strict borders. For example, School 1 in this study, which was described as being in a rural area, may be described as being in a semi/peri-urban area by a different author. Similarly, Schools 2–4, which were discussed as urban, may also be described as semi/peri-urban by a different author. It is therefore important to interpret the data cautiously. Nonetheless, the thrust of the work was the income disparity between families living in the same locality and how it affects the nutritional status of children. In this respect, the classification of place of residence scarcely affects the interpretation of the data. Fourth, not quantitatively determining the nature of the snack foods, that is whether they were healthy snack foods or unhealthy snack foods, is a clear limitation of this study. The desire to keep the design simple and the knowledge (borne out of experience) that snack foods for children in the studied part of Nigeria is typically nutritionally poor biscuits and pastries may attenuate the impact of the above limitation. Fifth and finally, the use of fees charged by schools as an indicator of family income and walking to and from school as a proxy for physical activity may not pass for standard practices. They are nonetheless sensible and logical determinants and served the purpose for which they were used effectively.

This is the first study to the author’s knowledge that investigated the impact of SES on the nutritional status of children in Nigeria while considering income inequality within the same place of residence. This makes the study novel and its findings interesting and capable of eliciting the right debate (and possibly further studies) on this important subject, with a view to a better planning of nutrition intervention programs in communities that have uniformity in place of residence, but wide variations in individual family income.

## 5. Conclusions

This study investigated the impact of income inequality and the differences in the living standards of people living in the same urban place of residence on the nutritional status of children. The findings show that the urban-poor group of children is disproportionately affected by malnutrition as it cumulates indices of thinness—that are worse than in their urban-rich or rural counterparts—and indices of overweight/obesity—that are worse than their rural counterparts. This is despite the close proximity of both places of residence. Children from high SES families had fewer siblings, apparently engaged in less physical activity, and were clearly more overweight/obese than the others. This calls for more studies on this subject to better plan nutrition intervention programs that take into account differences in the needs of people living in the same place of residence.

## Figures and Tables

**Table 1:** Relevant characteristics of the children in the four schools studied.

	Height (cm)		Weight (kg)		BMI (kg/m^2^)		No. of siblings	
	Male	Female	Male	Female	Male	Female	Male	Female
School 1	132.3 ± 10.8 ^a^	132.2 ± 8.8 ^a^	29.0 ± 7.1 ^a^	29.1 ± 5.4 ^a^	16.3 ± 1.8 ^a^	16.5 ± 1.7 ^a^	5 ± 2 ^b^	5 ± 2 ^c^
School 2	130.9 ± 10.3 ^a^	132.4 ± 9.2 ^a^	27.6 ± 5.8 ^a^	28.7 ± 7.6 ^a^	16.0 ± 1.9 ^a^	15.7 ± 2.2 ^a^	3 ± 2 ^a^	4 ± 2 ^b^
School 3	132.2 ± 9.2 ^a^	133.0 ± 8.8 ^a^	31.5 ± 8.4 ^ab^	33.8 ± 8.8 ^b^	17.8 ± 3.1 ^b^	18.9 ± 3.4 ^b^	3 ± 1 ^a^	3 ± 2 ^ab^
School 4	133.6 ± 11.6 ^a^	132.0 ± 8.4 ^a^	33.2 ± 10.0 ^b^	31.8 ± 10.3 ^ab^	18.3 ± 3.4 ^b^	18.3 ± 2.8 ^b^	2 ± 1 ^a^	2 ± 1 ^a^

Values followed by the same letter within each column are statistically similar (*p* > 0.05). The number of children in the schools are: School 1, 135 (48.9% females); School 2, 54 (40.7% females); School 3, 151 (51.0% females); and School 4, 120 (44.2% females). There were no cases of missing data.
